# Assessment of prognostic stratification in colorectal cancer liver metastases using preoperative CT habitat imaging

**DOI:** 10.3389/fonc.2026.1829205

**Published:** 2026-08-12

**Authors:** Hailong Feng, Mingmei Xue, Yuhua He, Gaiyun Zhang, Linshuai Xing

**Affiliations:** 1 Department of Colorectal and Anal Surgery, the First Affiliated Hospital of Henan Medical University, Weihui, China; 2 Department of Radiology, the First Affiliated Hospital of Henan Medical University, Weihui, China; 3 Department of Magnetic Resonance Imaging (MRI), the First Affiliated Hospital of Henan Medical University, Weihui, China

**Keywords:** colorectal cancer liver metastases, computed tomography, habitat imaging, prognostic stratification, radiomics, tumor heterogeneity

## Abstract

**Objective:**

This study aims to explore the value of radiomic features derived from preoperative CT habitat imaging in prognostic stratification of patients with colorectal cancer liver metastases (CRLM), and to construct the predictive model incorporating clinical information for individualized risk assessment.

**Methods:**

Retrospectively data from 196 patients with CRLM who underwent surgical resection were collected, including preoperative portal venous phase CT images and clinical information. First, tumor regions were segmented into habitat subregions using the K-means clustering algorithm to capture intra-tumoral heterogeneity. Subsequently, habitat radiomic features were extracted and dimensionality was reduced through Spearman correlation analysis followed by Least absolute shrinkage and selection operator regression, key features were then selected using the maximum relevance minimum redundancy method. Habitat models and combined clinical-habitat models were constructed based on Naive Bayes, multilayer perceptron (MLP), and XGBoost algorithms, respectively. Model performance was evaluated using receiver operating characteristic curves (ROC), decision curve analysis (DCA), and calibration curves.

**Results:**

Compared with Naive Bayes and MLP, XGBoost demonstrated superior predictive performance in evaluating the prognosis of patients with CRLM. In both the training and validation cohorts, the combined clinical-habitat model achieved the best performance, with the AUC of 0.935 and 0.893, the sensitivity of 95.00% and 84.00%, and the specificity of 94.87% and 84.85%, respectively. Decision curve analysis showed that the predictive model provided a high net clinical benefit, and calibration curves confirmed good agreement between the predicted probabilities and the actual observed outcomes. The proposed model presents significant advantages in predictive accuracy compared with previous studies based only on traditional radiomics or clinical indicators.

**Conclusion:**

Habitat imaging based on preoperative CT can effectively quantify intratumoral heterogeneity. The combined model constructed with machine learning algorithms exhibits excellent performance in prognostic stratification of patients with CRLM. This approach provides a non-invasive, high-precision novel tool for clinical prognostic evaluation, with important promotion value and promising application prospects.

## Introduction

Colorectal cancer (CRC) is one of the most common malignant tumors worldwide, with incidence and mortality rates ranking among the highest globally (1, 2). Liver metastases represent the leading cause of death in patients with CRC, approximately 50% of CRC patients develop liver metastases during the course of their disease, and 20% - 25% already have synchronous liver metastases at initial diagnosis (3, 4). Although recent advances in surgical techniques, optimization of chemotherapy regimens, and the application of targeted and immunotherapies have significantly improved outcomes for a subset of patients, colorectal cancer liver metastases (CRLM) exhibit substantial inter-patient heterogeneity, resulting in considerable variability in clinical outcomes (5, 6).

Accurate prognostic stratification is critical for individualized treatment decision-making in patients with CRLM. It not only helps identify high-risk patients for intensified therapeutic strategies but also avoids overtreatment and related toxicities in low-risk patients. Currently, most clinically used prognostic evaluation models rely on traditional clinicopathological factors, e.g., tumor number, size, CEA level, lymph node status of the primary lesion, extrahepatic metastasis, or simple radiological descriptions (7). However, these conventional approaches often fail to comprehensively capture the complex biological characteristics of tumors and the heterogeneity of their microenvironments, resulting in limited accuracy of prognostic prediction and difficulty in meeting the requirements of the precision medicine era.

CT has become the preferred imaging modality for preoperative assessment of CRLM due to its speed, widespread availability, high spatial resolution, and ability to provide rich morphological and partial functional information. It is widely used for lesion detection, localization, lesion number enumeration, size measurement, and resectability evaluation (8, 9). Traditional CT interpretation primarily relies on the subjective visual assessment and qualitative description by radiologists, which often falls short in capturing subtle intra-tumor heterogeneity (ITH) and exploring underlying biological information. Radiomics, by extracting high-dimensional features from CT images, has demonstrated potential in predicting various biological behaviors and clinical outcomes of tumors (10). However, tumors exhibit significant heterogeneity across patients, between tumors, and even within a single tumor. This heterogeneity is regulated by a combination of intrinsic and extrinsic factors, including the tumor microenvironment and genetic variations. Crucially, ITH plays a vital role in prognostic assessment, treatment response prediction, recurrence risk stratification, and drug resistance mechanism research in CRLM (11). Traditional radiomics analyses often focus on extracting average features from the whole tumor region, frequently neglecting the detailed characterization of ITH. This limitation, to some extent, constrains their accuracy and reliability in key clinical tasks for CRLM, such as molecular subtype classification and precise prognostic prediction. With the continuous advancement of artificial intelligence and deep learning, the field of medical radiomics has introduced habitat analysis, known as habitat imaging. This is a radiomics analysis method based on biological environmental characteristics. It describes and quantifies the heterogeneity within tissues and the relationships between different regions by extracting and analyzing features from medical imaging data. This method holds significant importance in tumor diagnosis, treatment decision-making, and prognosis assessment (12, 13). Therefore, this study aims to utilize habitat imaging technology to extract features from preoperative CT images of resected liver metastases. Furthermore, it seeks to construct the predictive model using machine learning algorithms for the precise quantification of prognosis in patients with CRLM, with the goal of providing new strategies for related clinical diagnosis and treatment.

## Materials and methods

### Patient collective

**Figure 1:**
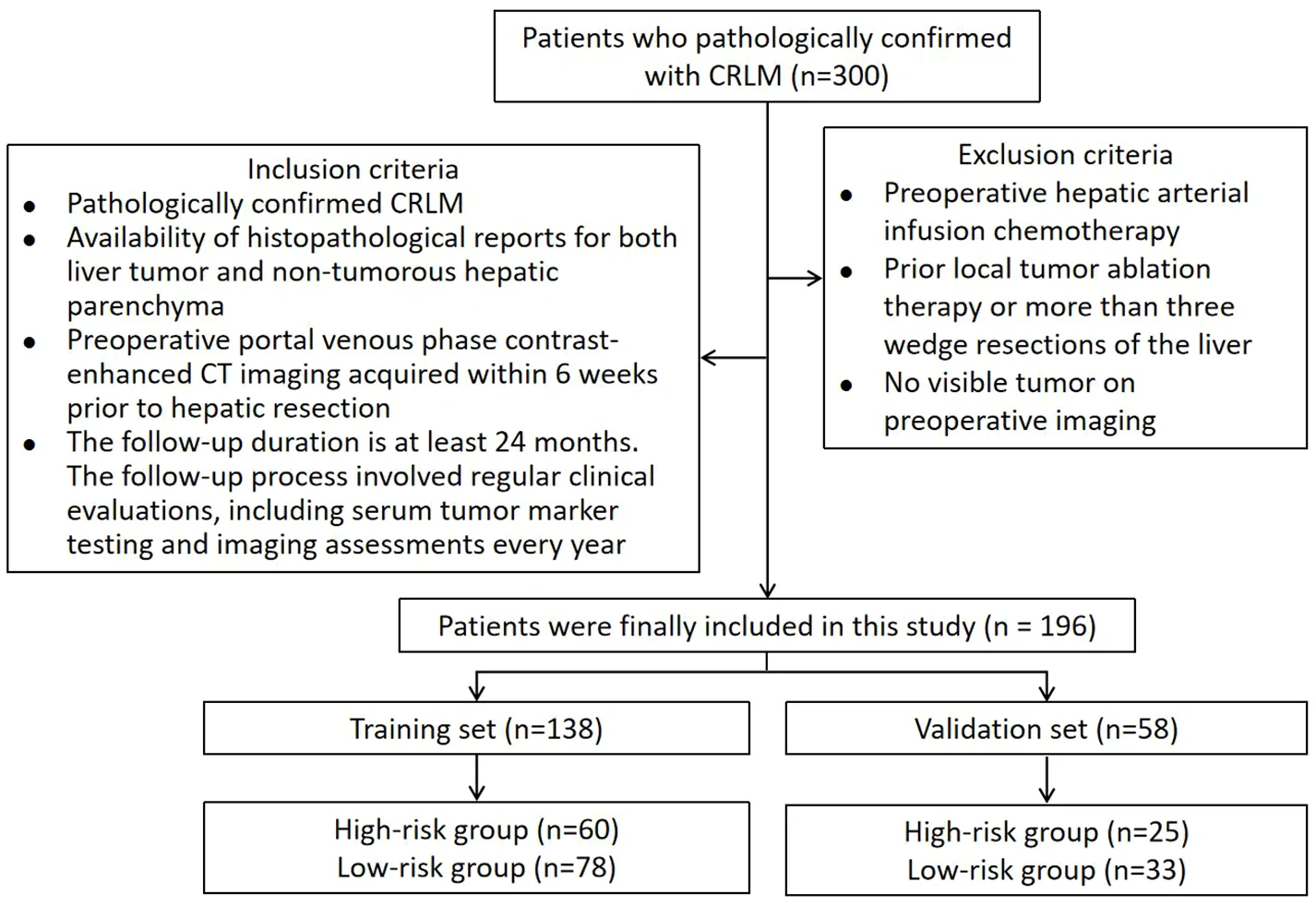
Subject selection flowchart for this research.

This study was a retrospective study and was approved by the relevant ethics committee. Inclusion criteria: 1). Pathologically confirmed CRLM; 2) Availability of histopathological reports for both liver tumor and non-tumorous hepatic parenchyma; 3) Preoperative portal venous phase contrast-enhanced CT imaging acquired within 6 weeks prior to hepatic resection; 4) The follow-up duration is at least 24 months. The follow-up process involved regular clinical evaluations, including serum tumor marker testing and imaging assessments every year. Exclusion criteria: 1). Preoperative hepatic arterial infusion chemotherapy; 2) Prior local tumor ablation therapy or more than three wedge resections of the liver; 3) No visible tumor on preoperative imaging. A total of 196 patients were finally included in the study. Among them, patients who experienced recurrence of the primary and/or metastatic lesions or died were assigned to the high-risk group, whereas those without recurrence of the primary and/or metastatic lesions were classified as the low-risk group (**Figure 1**). Using stratified sampling, the cohort was divided into a training set of 138 cases and a validation set of 58 cases at a 7:3 ratio. Clinical data were recorded for each patient, including sex, age, body mass index, colon primary at operation, more multiple metastases, max tumor size, chemo before liver surgery and receive portal vein embolization before surgery (**Table 1**). In addition, pathological assessments provided the percentages of tumor necrosis, tumor fibrosis, and tumor mucin.

**Table 1:** Clinical characteristics of CRLM patients in the training and validation sets.

Variable	Training set		*P* value	Validation set		*P* value	z/ value	*P* value (Training vs Validation)
	Low-risk group (n = 78)	High-risk group (n = 60)		Low-risk group (n = 33)	High-risk group (n = 25)			
Age (years)	61.06 ± 10.99	59.1 ± 13.39	0.643	58.61 ± 12.14	58.52 ± 13.99	0.980	-0.850	0.395
Gender			0.439			0.258	0.046	0.830
Male	48 (61.54 %)	33 (55.00 %)		22 (66.67 %)	13 (52.00 %)			
Female	30 (38.46 %)	27 (45.00 %)		11 (33.33 %)	12 (48.00 %)			
Body mass index (kg/ m^2^)	28.19 ± 5.51	26.62 ± 4.51	0.074	27.69 ± 4.16	25.85 ± 4.60	0.079	-0.850	0.395
Colon primary at operation			0.454			0.366	2.359	0.125
Yes	44 (56.41 %)	30 (50.00 %)		20 (60.61 %)	18 (72.00 %)			
No	34 (43.59 %)	30 (50.00 %)		13 (39.39 %)	7 (28.00 %)			
More multiple metastases			0.499			0.729	1.831	0.176
Yes	41 (52.56 %)	35 (58.33 %)		21 (63.64 %)	17 (68.00 %)			
No	37 (47.44 %)	25 (41.67 %)		12 (36.36 %)	8 (32.00 %)			
Max tumor size (cm)	3.29 ± 3.00	3.86 ± 2.23	0.008	3.08 ± 1.99	3.70 ± 2.56	0.550	-0.338	0.735
Chemo before liver surgery			0.439			0.034	0.127	0.722
Yes	47 (60.26 %)	40 (66.67 %)		16 (48.48 %)	19 (76.00 %)			
No	31 (39.74 %)	20 (33.33 %)		17 (51.52 %)	6 (24.00 %)			
Receive portal vein embolization before surgery			0.818			0.375	9.070	0.003
Yes	6 (7.69 %)	4 (6.67 %)		6 (18.18 %)	7 (28.00 %)			
No	72 (92.31 %)	56 (93.33 %)		27 (81.82 %)	18 (72.00 %)			
Percentage of tumor necrosis (%)	28.78 ± 22.81	30.50 ± 22.79	0.647	36.21 ± 23.25	32.80 ± 29.44	0.360	-1.154	0.248
Percentage of tumor fibrosis (%)	19.23 ± 23.30	18.42 ± 14.34	0.159	19.09 ± 23.33	15.20 ± 19.17	0.330	-1.39	0.165
Percentage of tumor mucin (%)	8.21 ± 20.73	1.75 ± 6.50	0.052	0.15 ± 0.87	7.80 ± 13.62	0.002	-0.246	0.805

### Imaging protocols

All patients in this study underwent routine contrast-enhanced CT scans adhering to established imaging protocols. Abdominal imaging was performed using multidetector CT scanners (Lightspeed 16 and VCT; GE Healthcare, Madison, WI, USA). The primary acquisition parameters included: automatic tube current modulation (220–380 mA), noise index (12 - 14), gantry rotation time (0.7 - 0.8 seconds), and the scan delay of 80 seconds. The contrast agent used was Iohexol (350 mgI/mL), administered intravenously at a dose of 1.3 mL/kg body weight with an injection rate of 3.0 mL/s. For patients who received neoadjuvant chemotherapy, postoperative CT images were utilized. In patients who underwent preoperative portal vein embolization (PVE), pre-PVE CT scan images were used.

### Image segmentation

**Figure 2:**
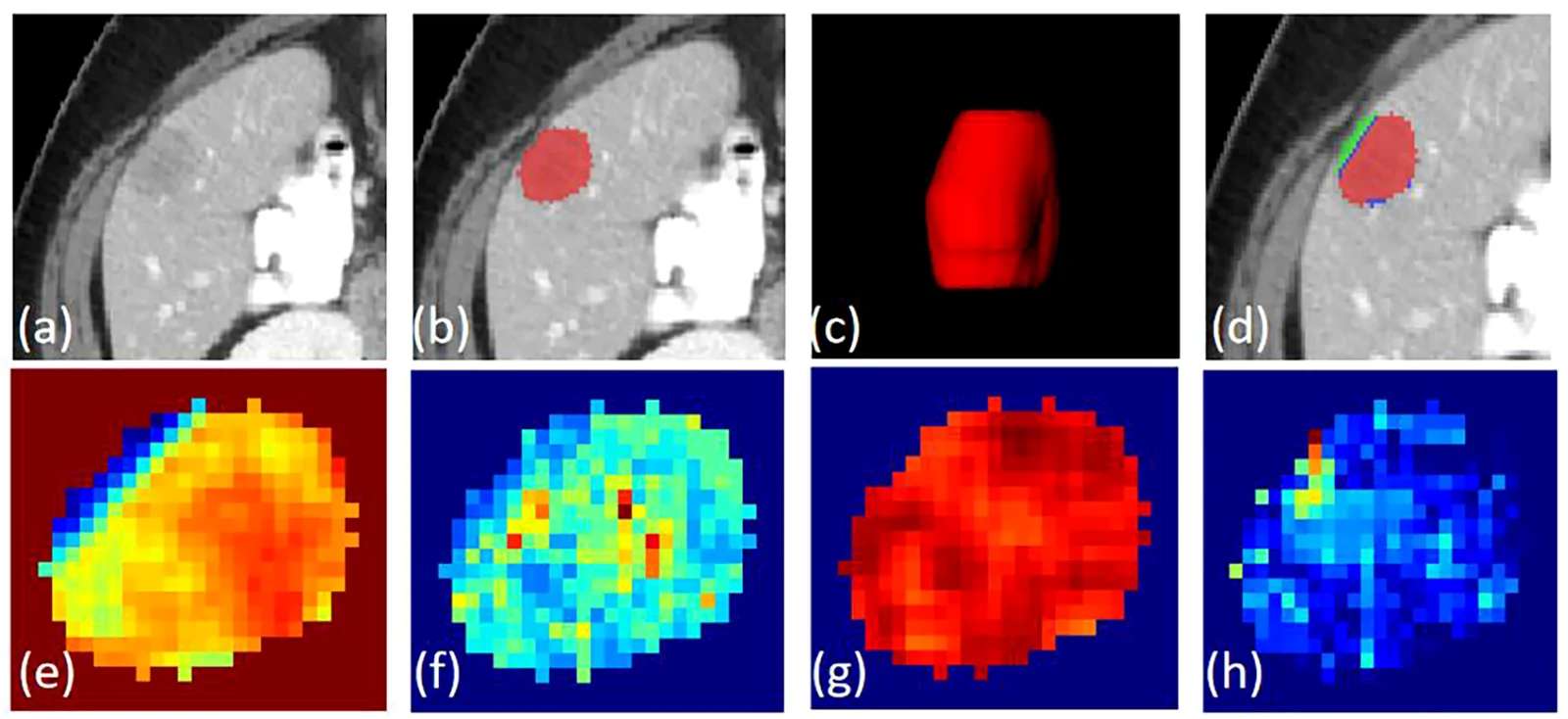
Male, 69 years old, CRLM, high-risk group, ITH score 0.89, indicating high intratumoral heterogeneity. **(A)** CT image; **(B)** Image segmentation result; **(C)** Lesion; **(D)** Single-slice subregion generation results, with red representing h1 subregion, green representing h2 subregion and blue representing h3 subregion; **(E–H)** Habitat characteristics visualization.

Portal venous phase CT images were imported into ITK‐SNAP software. Regions of interest (ROIs) were delineated by two radiologists with 8 and 15 years of experience in abdominal imaging diagnosis, respectively. Any discrepancies in segmentation between the two readers were resolved by a chief radiologist who determined the final ROI boundaries. For patients with multiple lesions, the largest lesion was selected for segmentation. Inter-observer agreement of the ROI segmentation between the two readers was evaluated using the Dice similarity coefficient. The lesion segmentation procedure is shown in **Figures 2A–C**.

### Data preprocessing

Normalize the portal venous phase CT images by cropping them to a uniform size (512 × 512) and scaling the image intensity to a range of 0 - 1.

### Habitat sub-regions generation and radiomics analysis

To generate the tumor microenvironment atlas (14, 15) (**Figures 2E–H**), the unsupervised K-means clustering method was used to identify similar subregions within all tumor voxels. The number of clusters k was set from 3 to 10 to control the clustering resolution. The Calinski Harabasz (CH) index was then used as the criterion for selecting the optimal cluster number, with the final k determined by the maximum CH value. After clustering, pixels belonging to the same cluster were assigned the same color to generate a cluster label map (**Figure 2D**).

For radiomics analysis based on CT habitat imaging, we utilized the PyRadiomics software package (Version 2.20, https://github.com/Radiomics/pyradiomics) to extract features from the tumors. A total of 1197 features were extracted.

Intratumor heterogeneity was quantified using the ITH score, calculated as follows:


$$ITH_{score}=1-\frac{1}{S_{total}}\sum_{i=1}^{v}\frac{S_{i,\max}}{n_{i}}$$

The number of connected components is denoted as *n_i_*, and the maximum area of connected components within each cluster *i* is denoted as $S_{i,max}$. A greater number of connected components and a smaller maximum area per cluster correspond to a more heterogeneous pattern. Here, *v* represents the number of clusters in the labeled image, and $S_{total}$ denotes the total tumor area. The ITH score ranges from 0 to 1. A higher ITH score indicates a more scattered distribution of clusters, reflecting greater intra-tumoral heterogeneity.

### Feature selection and model construction

To reduce feature redundancy and extract key variables, we first performed correlation assessment for highly repetitive features using the Spearman correlation coefficient. Building upon this, the Least Absolute Shrinkage and Selection Operator (LASSO) algorithm was introduced to further screen for features with significant contributions. Key parameter settings for the LASSO algorithm were as follows: the regularization parameter α was set to 0.01, the maximum number of iterations max_iter was set to 1000, and the number of cross-validation folds n_folds was set to 5, ensuring model stability and generalization capability. Subsequently, the Max-Relevance and Min-Redundancy (mRMR) method was employed to rank and select features, from which a subset of the top 10 features achieving the optimal balance between relevance and independence was chosen. To enhance the interpretability of feature importance, the SHapley Additive exPlanations (SHAP) analysis method was further utilized to create visual charts, revealing the contribution levels of each feature from both global and local perspectives (16).

**Figure 3:**
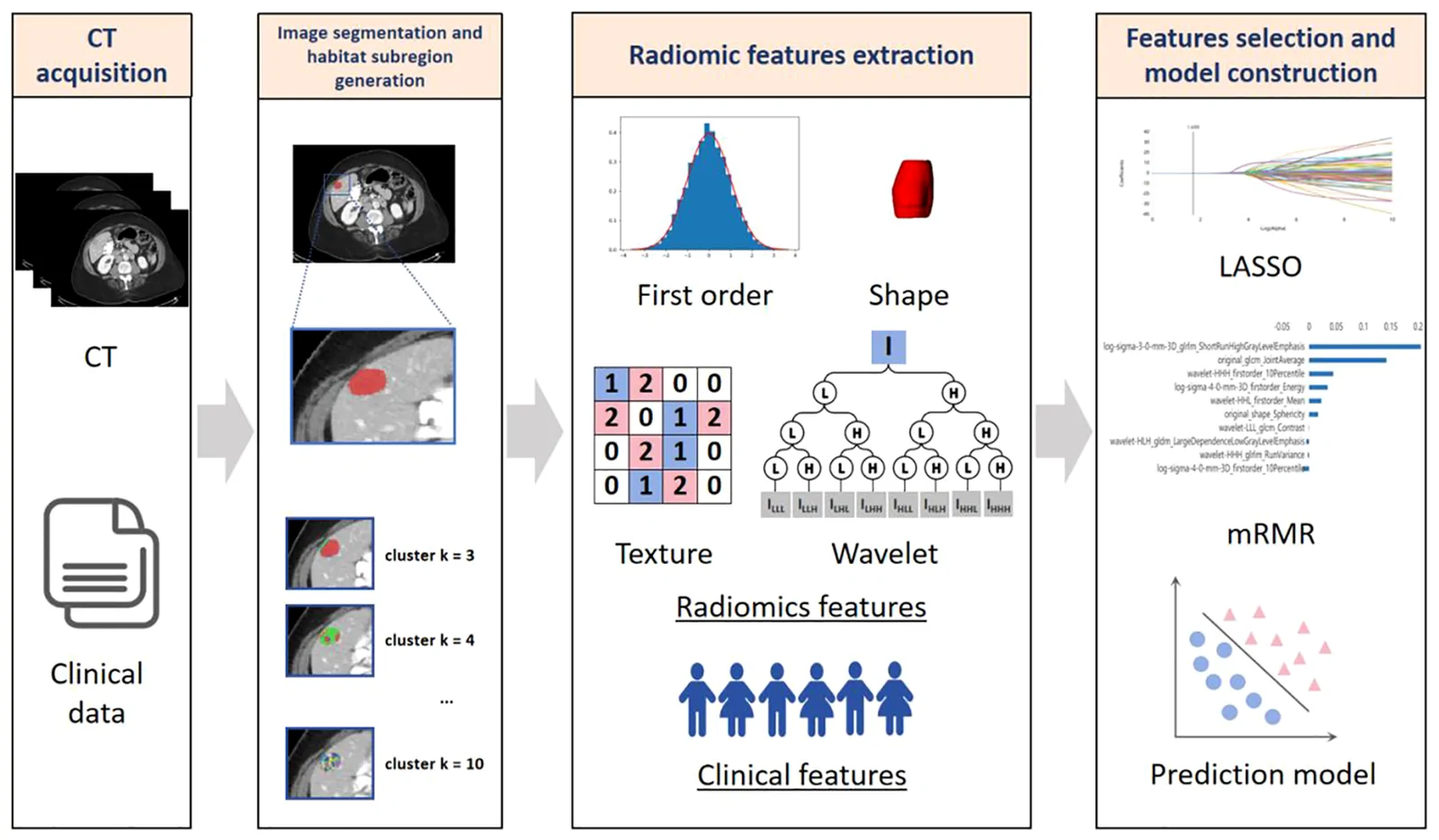
Habitat radiomics analysis workflow for predicting the prognostic stratification in CRLM patients.

Based on the habitat subregion features selected above, three algorithms, namely Naive Bayes (17), Multilayer Perceptron (MLP) (18), and XGBoost (19), were used to construct the habitat model, respectively. On this basis, clinical features were further integrated to build the combined clinical-imaging model, so as to improve the overall modeling performance and biological interpretability (**Tables 1**, **2**; **Figure 3**).

**Table 2:** The details of different classifiers.

Classifier	Parameters
NaiveBayes	Priors: None, var_smoothing: 1e-9
Multilayer perceptron	Activation: relu, hidden_layer_sizes: 128, solver: adam, alpha: 0.001, batch_size: 32, learning_rate_init: 0.001, max_iter: 300, random_state: 42
XGBoost	Learning_rate: 0.05, n_estimators: 200, max_depth: 4, subsample: 0.8, colsample_bytree: 0.8, gamma: 0, reg_alpha: 0.1, reg_lambda: 1, objective: binary, random_state: 42

### Statistical analysis

The sample size was determined based on the availability of eligible cases and the need for adequate representation of both risk groups. For comparisons of categorical data, the chi-square test was used; for continuous data, either the Mann Whitney U test or the independent samples t-test was applied, depending on data distribution. The performance of the prediction models was assessed by the area under the receiver operating characteristic curve (AUC). Model stability and clinical net benefit were evaluated using decision curve analysis (DCA) and calibration curves, respectively. All statistical analyses were performed using SPSS software (version 31.0, IBM), and the P value < 0.05 was considered statistically significant.

## Results

### Statistical analysis of clinical features

A total of 85 patients in the high-risk group with CRLM and 111 patients in the low-risk group were included. Regarding clinical characteristics, significant differences were observed between the two groups in terms of body mass index and max tumor size. Consequently, body mass index and max tumor size were incorporated into the predictive model as clinical indicators (**Table 1**). These two variables were selected due to their statistical significance, suggesting their potential important role in predicting CRLM prognosis. Including these clinical indicators can enhance the model’s ability to accurately predict prognosis by accounting for relevant patient-specific factors.

### Tumor internal heterogeneity analysis

The ITH score calculated from preoperative CT images showed a wide variation across all patients, ranging from 0.000 to 0.937, with a mean of 0.498 ± 0.265 and a median (interquartile range) of 0.517 (0.223, 0.758).

### Habitat radiomics feature selection

**Figure 4:**
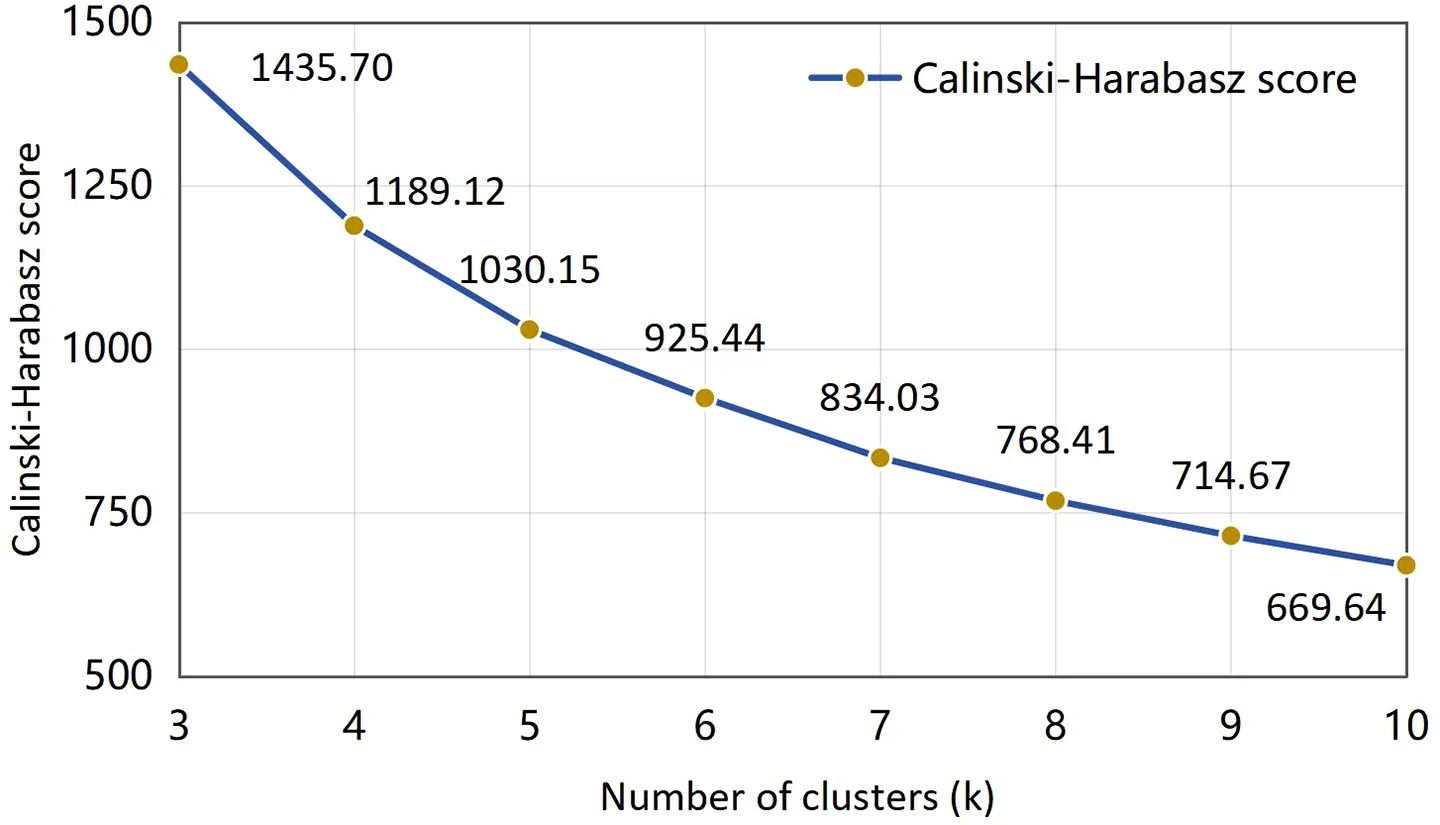
The Calinski-Harabasz value evaluates clustering results and selects the optimal number of clusters (k = 3), with the horizontal coordinate representing the number of clusters k and the vertical coordinate representing the CH value.

Clustering was performed using the K-means algorithm based on imaging features, with the number of cluster centers set from 3 to 10. The Calinski-Harabasz index was used as the evaluation metric, where a higher CH value indicated a better result. The optimal number of cluster centers was determined to be 3 (**Figure 4**). Corresponding subregions were generated, and the three habitat subregions were assigned distinct color labels.

**Figure 5:**
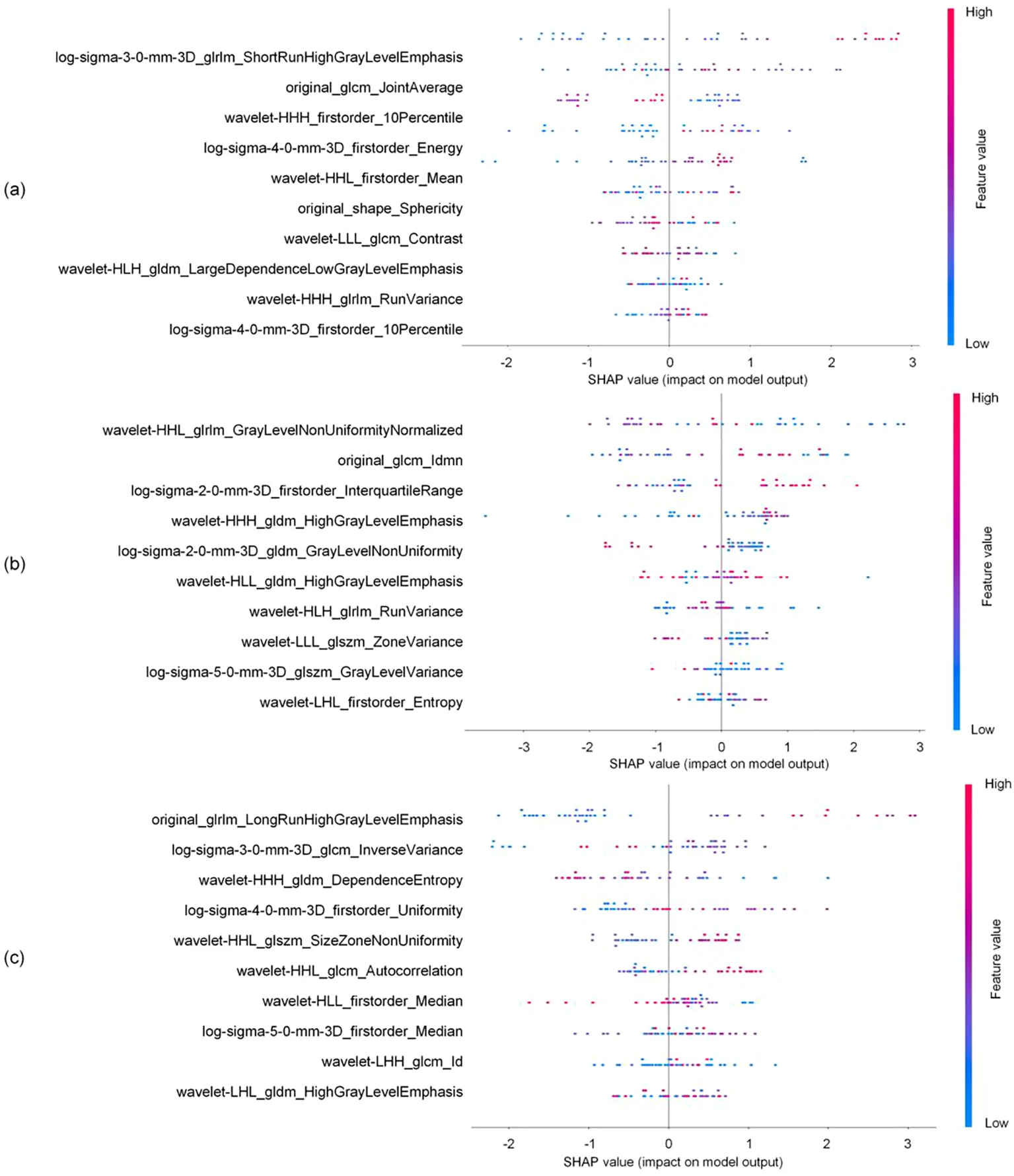
SHAP plots of the features most relevant to prognosis. **(A)** Results for habitat subregion h1; **(B)** Results for habitat subregion h2; **(C)** Results for habitat subregion h3.

For the habitat radiomics dataset, 1197 features were extracted from each of the three habitat subregions (h1, h2, and h3). After removing redundant features, 10 features were finally selected from each subregion, giving a total of 30 features for constructing the classification model. SHAP values were used to identify the most prognosis-relevant features in each subregion (**Figure 5**).

### Predictive performance

**Figure 6:**
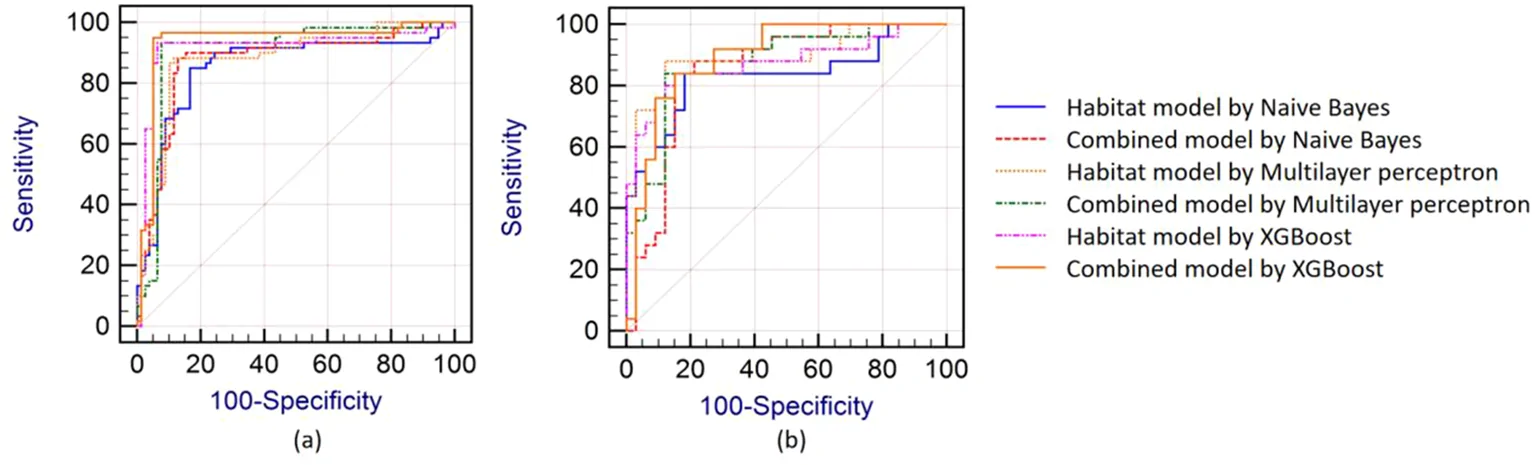
ROC of the predictive models constructed by different classifiers. **(A)** Training set; **(B)** Validation set.

For model construction, we established habitat models based on three mainstream algorithms Naive Bayes, MLP, and XGBoost, respectively. The combined models were further construction by integrating habitat features and clinical features. The results demonstrated that the combined model outperformed the single habitat model across multiple performance metrics, suggesting that the complementarity between clinical information and imaging features can significantly improve the accuracy of prognosis prediction. Among them, the XGBoost model showed superior predictive performance and robustness due to its advantages in handling nonlinear relationships and feature interactions, with a training set AUC of 0.935 (95% CI: 0.880 - 0.970) and a validation set AUC of 0.893 (95% CI: 0.784 - 0.959). The MLP also exhibited promising potential in complex pattern recognition, with a training set AUC of 0.904 (95% CI: 0.842 - 0.948) and a validation set AUC of 0.869 (95% CI: 0.755 - 0.943). In comparison, although the Naive Bayes model had the merits of high interpretability and computational efficiency, it was limited in fitting complex data structures, with a training set AUC of 0.872 (95% CI: 0.805 - 0.923) and a validation set AUC of 0.850 (95% CI: 0.731 - 0.930). Improved versions incorporating prior knowledge may be considered in future studies (**Table 3**; **Figure 6**).

**Table 3:** Performance metrics of the predictive models constructed by different classifiers.

Group	Algorithm	Model	AUC (95% confidence interval)	Sensitivity (%)	Specifcity (%)	Standard error
Training set	NaiveBayes	Habitat model	0.851 (0.781 - 0.906)	85.00	83.33	0.0369
		Combined model	0.872 (0.805 - 0.923)	88.33	87.18	0.0336
	Multilayer perceptron	Habitat model	0.878 (0.811 - 0.927)	88.33	88.46	0.0316
		Combined model	0.904 (0.842 - 0.948)	93.33	92.31	0.0304
	XGBoost	Habitat model	0.918 (0.860 - 0.958)	93.33	93.59	0.0301
		Combined model	0.935 (0.880 - 0.970)	95.00	94.87	0.0263
Validation set	NaiveBayes	Habitat model	0.832 (0.710 - 0.917)	84.00	81.82	0.0597
		Combined model	0.850 (0.731 - 0.930)	84.00	84.85	0.0531
	Multilayer perceptron	Habitat model	0.862 (0.746 - 0.938)	84.00	84.85	0.0525
		Combined model	0.869 (0.755 - 0.943)	84.00	87.88	0.0484
	XGBoost	Habitat model	0.874 (0.760 - 0.947)	84.00	84.85	0.0518
		Combined model	0.893 (0.784 - 0.959)	84.00	84.85	0.0428

## Discussion

**Figure 7:**
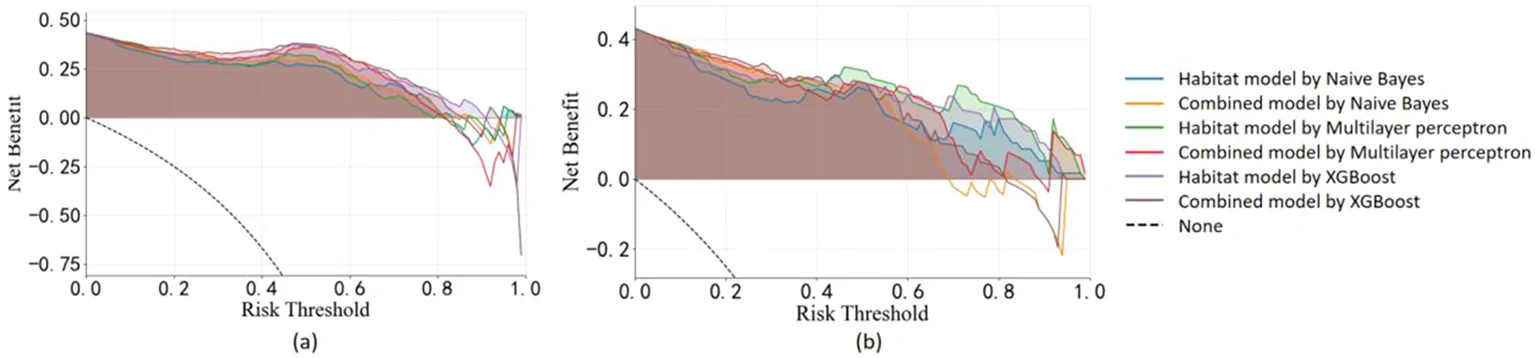
DCA of the predictive models constructed by different classifiers. **(A)** Training set; **(B)** Validation set.

**Figure 8:**
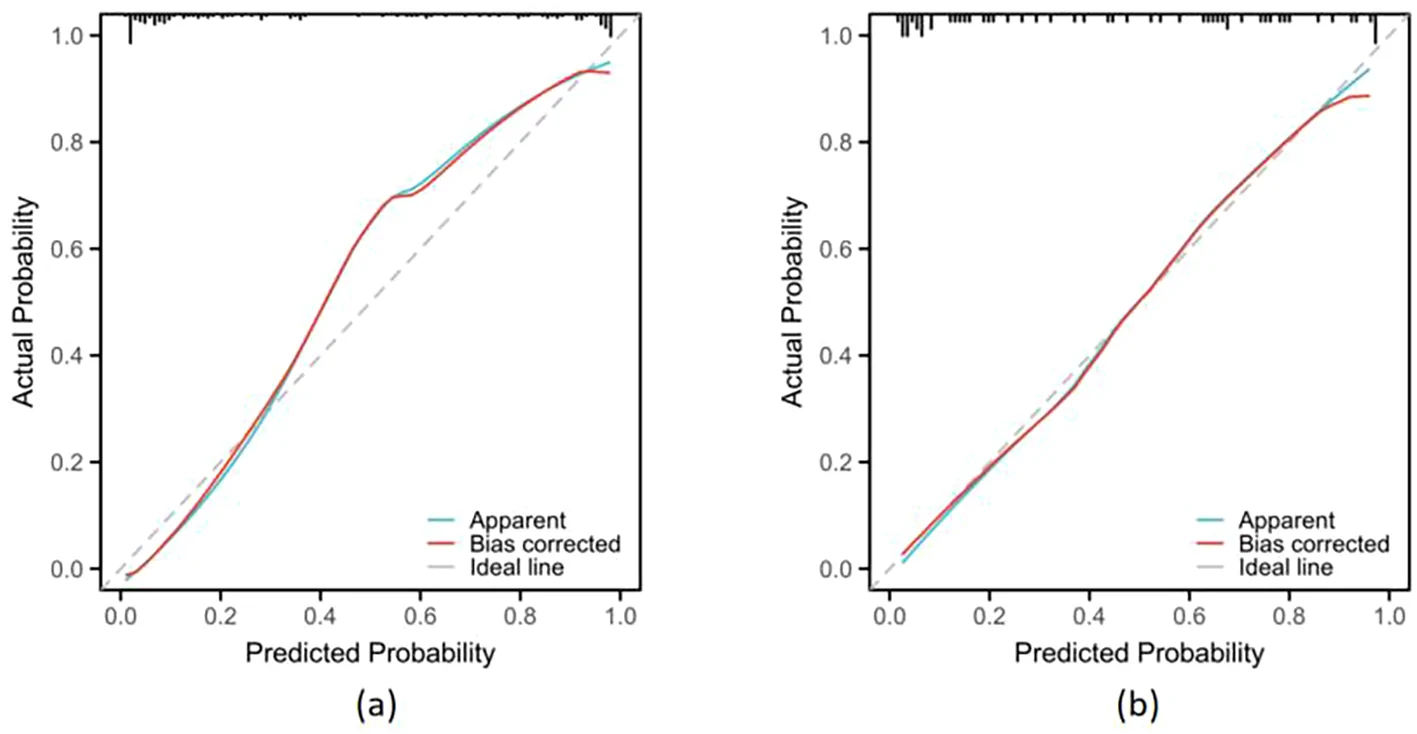
Calibration curve of the combined model constructed by XGBoost. **(A)** Training set; **(B)** Validation set.

Based on the preoperative clinical data and portal venous phase CT images of patients with CRLM, this study first extracted habitat imaging features from liver metastases. Subsequently, by integrating clinical information, habitat models and combined clinical-habitat models were constructed using the Naive Bayes, MLP, and XGBoost algorithms, respectively. Their application value in prognostic assessment for CRLM patients was systematically explored. The results showed that, both in the training and validation sets, the combined clinical-habitat model demonstrated the best diagnostic performance. The AUCs were 0.935, 0.904, 0.872 and 0.893, 0.869, 0.850, respectively, with optimal sensitivity reaching 95.00% and 84.00%, and optimal specificity reaching 94.87% and 87.88%. Furthermore, DCA confirmed that all models could provide high clinical net benefit for patients across different threshold probabilities, demonstrating good practicality and promising potential for generalization (**Figure 7**). The calibration curve of the combined model built with XGBoost was close to the ideal diagonal, indicating good consistency between predicted probabilities and actual observed outcomes (**Figure 8**). The key strengths of XGBoost lie in two main aspects. First, it incorporates both L1 and L2 regularization terms directly into the objective function, effectively controlling model complexity and preventing overfitting. This characteristic is particularly critical in radiomics research, where the feature dimension is typically high yet the sample size is relatively limited. Second, the relationships between radiomic features and clinical outcomes are often characterized by complex non-linearity. XGBoost utilizes a hierarchical splitting structure within decision trees to automatically capture high-order interaction effects among features without requiring predefined functional forms. This grants the model a natural advantage when processing complex imaging phenotypes, such as texture heterogeneity and multi-scale filtered features.

In recent years, artificial intelligence technology has advanced rapidly in the field of medical image analysis, offering novel perspectives for precision diagnosis and treatment of tumors, owing to its advantages of being non-invasive, high-throughput, and widely accessible (20). In the realm of radiomics research on CRLM, several studies have attempted to apply unsupervised learning methods for feature extraction and risk stratification from preoperative CT images. For example, models built upon unsupervised radiomics and machine learning for prognostic prediction in CRLM patients achieved an AUC of 0.60 - 0.64 for 6-month survival prediction (21) and an AUC of 0.66 for 5-year survival prediction (22). Another study predicted CRLM prognosis using CT and magnetic resonance imaging radiomic features and reported an accuracy as high as 97% (23). However, the sample size was small, limiting the reliability of the findings. Furthermore, a study employing a machine-learning-derived tumor burden classification method determined optimal cutoff values for the number of liver metastases and the diameter of the largest lesion to predict 5-year overall survival (24). Cohen’s d values between the low-, intermediate-, and high-risk groups were 2.73, 1.61, and 0.84, respectively, demonstrating a certain degree of discriminative ability. Nevertheless, the approach used to determine the optimal cutoff values for metastasis count and maximum tumor size could affect model performance.

Compared with previous studies, the innovation of this research lies in being the first to deeply integrate habitat imaging technology with multiple mainstream machine learning algorithms, constructing the combined clinical-habitat prediction model. This achieves a leap in assessment from macroscopic morphology to microscopic heterogeneity. Traditional radiomics typically treats tumors as homogeneous entities, extracting only overall texture or intensity features, which makes it difficult to capture the complex biological heterogeneity within the tumor. In contrast, this study employs spatial segmentation technology to identify habitat sub-regions with different imaging characteristics, quantitatively characterizing ITH. By combining methods such as Spearman correlation, LASSO regression, and mRMR to select key features, the ultimately established combined model achieved AUCs of 0.935 and 0.893 in the training and validation sets, respectively. This performance is significantly superior to that of previously reported radiomics models. This result indicates that, compared to traditional whole image analysis or single-load indicator classification, the strategy based on habitat imaging can more comprehensively and deeply reveal the biological complexity within tumors, thereby providing more precise decision support for prognosis prediction.

Among the selected key radiomic features, high values of GLRLM-based features such as log-sigma-3-0-mm-3D_glrlm_ShortRunHighGrayLevelEmphasis and original_glrlm_LongRunHighGrayLevelEmphasis, as well as the GLDM-based feature wavelet-HHH_gldm_HighGrayLevelEmphasis, indicate the presence of regions with elevated density or increased enhancement within the tumor. Notably, all three habitat subregions (h1, h2, and h3) encompass high-gray-level-related features, suggesting that each subregion contributes to capturing active tumor areas, albeit with different scale preferences. Specifically, h1 tends to emphasize short-run patterns (refining fine textures), whereas h3 includes long-run patterns (reflecting macroscopic structures), thereby demonstrating complementary multi-scale information. Furthermore, features such as Contrast, RunVariance, ZoneVariance, GrayLevelNonUniformity, Entropy, and SizeZoneNonUniformity are associated with inter-pixel gray-level differences, textural roughness, and subregional distribution patterns within the tumor. In a previous study predicting response to oxaliplatin-based chemotherapy in CRLM patients, texture heterogeneity features including GLCM Contrast, GLSZM SizeZoneNonUniformityNormalized, and GLRLM RunLengthNonUniformityNormalized were similarly identified as key predictors by the decision tree model (25), underscoring the clinical relevance of these heterogeneity metrics. In addition, Sphericity reflects tumor morphological irregularity and infiltrative growth patterns. In a CRLM cohort treated with bevacizumab, Sphericity was found to be significantly correlated with rim enhancement on portal venous phase imaging, and patients with rim enhancement exhibited significantly shorter overall survival (26). These findings indicate that reduced Sphericity is directly associated with aggressive biological phenotypes and poor prognosis. Moreover, in multiple machine learning models for predicting chemotherapy response in CRLM, shape_Sphericity was consistently selected as a key feature (25), further validating its prognostic value.

Habitat imaging and conventional radiomics both originate from the quantitative analysis of medical images, with the goal of extracting deep-level features for disease diagnosis, treatment response assessment, and prognosis prediction. However, they differ fundamentally in concept and technical approach. Conventional radiomics typically treats the tumor as a structurally homogeneous entity, extracting macroscopic statistical features that fail to capture the biological complexity within the tumor. In contrast, habitat imaging emphasizes the spatial heterogeneity of the intratumoral microenvironment, partitioning the tumor into multiple habitat subregions with distinct textures, densities, or enhancement patterns, thereby revealing the spatial distribution characteristics of ITH and its role in tumor progression (27–29). Currently, commonly used habitat segmentation methods include K-means clustering, Gaussian mixture models, and the OTSU algorithm, among which K-means clustering is widely adopted due to its simplicity and efficiency. Furthermore, based on the segmented habitat model, an ITH score can be computed to quantitatively describe the degree of intratumoral heterogeneity. Previous studies have indicated that different molecular subtypes or phenotypes of tumors may exhibit varying levels of ITH, influenced by epigenetic regulation or transcriptomic variations (13, 30). ITH is not only closely associated with tumor diagnosis, but also profoundly affects treatment sensitivity, therapeutic response, recurrence monitoring, drug resistance, and ultimately overall prognosis (31). Clinically, high heterogeneity is often associated with stronger proliferation, invasion, and metastatic potential, leading to poor treatment response and unfavorable prognosis (32). The advantage of the ITH score lies in its ability to noninvasively and quantitatively characterize tumor heterogeneity from routine imaging. In addition to calculating the ITH score, this study also performed systematic feature extraction and modeling analysis on different habitat subregions. The results showed that the habitat imaging model achieved significant efficacy in prognosis prediction, further validating the application potential of habitat radiomics in precision medicine.

However, this study has several limitations. First, the retrospective data were obtained from a relatively concentrated source, and the sample size was comparatively limited, which may affect the external generalizability of the model. Our future work will focus on external validation and prospective testing to confirm the model’s utility across diverse clinical settings. Second, while this study stratified patients into high- and low-risk groups for prognostic prediction, it lacked comprehensive clinical survival data, such as overall survival. In future work, we will incorporate more complete survival metrics to further validate the model’s predictive value for long-term survival outcomes. Meanwhile, we will standardize the collection of preoperative serum tumor markers, e.g., CEA, CA19-9, CA125, AFP to evaluate their complementary value alongside habitat radiomics features, thereby constructing comprehensive prognostic models that integrate multidimensional information. Third, the habitat subregions in this research were defined based on clustering algorithms, lacking well-justified biological interpretability. Future work should incorporate pathological and genomic analyses to further elucidate the biological significance of these habitat subregions.

## Conclusions

In conclusion, this study demonstrates that preoperative CT-based habitat imaging can effectively quantify intratumoral heterogeneity, and when combined with advanced algorithms such as XGBoost, enables the construction of high performance prognostic models, thereby providing a feasible pathway for precise prognostic stratification of patients with CRLM.

## Data Availability

The raw data supporting the conclusions of this article will be made available by the authors, without undue reservation.

## References

[1] Dekker E Tanis PJ Vleugels JLA Kasi PM Wallace MB . Colorectal cancer. Lancet. (2019) 394:1467–80. doi: 10.1016/S0140-6736(19)32319-0 31631858

[2] Abedizadeh R Majidi F Khorasani HR Abedi H Sabour D . Colorectal cancer: a comprehensive review of carcinogenesis, diagnosis, and novel strategies for classified treatments. Cancer Metastasis Rev. (2024) 43:729–53. doi: 10.1007/s10555-023-10158-3 38112903

[3] Shin AE Giancotti FG Rustgi AK . Metastatic colorectal cancer: mechanisms and emerging therapeutics. Trends Pharmacol Sci. (2023) 44:222–36. doi: 10.1016/j.tips.2023.01.003 36828759 PMC10365888

[4] Cañellas-Socias A Sancho E Batlle E . Mechanisms of metastatic colorectal cancer. Nat Rev Gastroenterol Hepatol. (2024) 21:609–25. doi: 10.1038/s41575-024-00934-z 38806657

[5] Patel RK Rahman S Schwantes IR Bartlett A Eil R Farsad K . Updated management of colorectal cancer liver metastases: scientific advances driving modern therapeutic innovations. Cell Mol Gastroenterol Hepatol. (2023) 16:881–94. doi: 10.1016/j.jcmgh.2023.08.012 37678799 PMC10598050

[6] Griffiths CD Karanicolas P Gallinger S Wei AD Francescutti V Serrano PE . Health-related quality of life following simultaneous resection for synchronous colorectal cancer liver metastases. Ann Surg Oncol. (2023) 30:1331–8. doi: 10.1245/s10434-022-12696-6 36350458 PMC11005481

[7] Dueland S Smedman TM Syversveen T Grut H Hagness M Line PD . Long-term survival, prognostic factors, and selection of patients with colorectal cancer for liver transplant: a nonrandomized controlled trial. JAMA Surg. (2023) 158:e232932. doi: 10.1001/jamasurg.2023.2932 37494056 PMC10372758

[8] Xing Q Cui Y Liu M Gu XL Li XT Xing BC . Preoperative CT-based morphological heterogeneity for predicting survival in patients with colorectal cancer liver metastases after surgical resection: a retrospective study. BMC Med Imaging. (2024) 24:343. doi: 10.1186/s12880-024-01524-w 39696033 PMC11657693

[9] Wesdorp NJ Kemna R Bolhuis K van Waesberghe JHTM Nota IMGC Struik F . Interobserver variability in CT-based morphologic tumor response assessment of colorectal liver metastases. Radiol Imaging Cancer. (2022) 4:e210105. doi: 10.1148/rycan.210105 35522139 PMC9152692

[10] Inchingolo R Maino C Cannella R Vernuccio F Cortese F Dezio M . Radiomics in colorectal cancer patients. World J Gastroenterol. (2023) 29:2888–904. doi: 10.3748/wjg.v29.i19.2888 37274803 PMC10237092

[11] Urh K Zidar N Tomažič A Boštjančič E . Intra-tumor heterogeneity of cancer stem cell-related genes and their potential regulatory microRNAs in metastasizing colorectal carcinoma. Oncol Rep. (2022) 48:193. doi: 10.3892/or.2022.8408 36111489

[12] Zhang X Su GH Chen Y Gu YJ You C . Decoding intratumoral heterogeneity: clinical potential of habitat imaging based on radiomics. Radiology. (2023) 309:e232047. doi: 10.1148/radiol.232047 38085080

[13] Huang H Chen H Zheng D Chen C Wang Y Xu L . Habitat-based radiomics analysis for evaluating immediate response in colorectal cancer lung metastases treated by radiofrequency ablation. Cancer Imaging. (2024) 24:44. doi: 10.1186/s40644-024-00692-w 38532520 PMC10964536

[14] de Visser KE Joyce JA . The evolving tumor microenvironment: From cancer initiation to metastatic outgrowth. Cancer Cell. (2023) 41:374–403. doi: 10.1016/j.ccell.2023.02.016 36917948

[15] Yin X Sha H Cao X Ge X Li T Cui Y . Tumor habitat-derived radiomics features in pretreatment CT scans for predicting concurrent chemoradiotherapy responses in nasopharyngeal carcinoma: a retrospective study. Quant Imaging Med Surg. (2025) 15:2917–28. doi: 10.21037/qims-24-1642 40235772 PMC11994483

[16] Yang X Qiu H Wang L Wang X . Predicting colorectal cancer survival using time-to-event machine learning: retrospective cohort study. J Med Internet Res. (2023) 25:e44417. doi: 10.2196/44417 37883174 PMC10636616

[17] Ateeq T Faheem ZB Ghoneimy M Ali J Li Y Baz A . Naïve Bayes classifier assisted automated detection of cerebral microbleeds in susceptibility-weighted imaging brain images. Biochem Cell Biol. (2023) 101:562–73. doi: 10.1139/bcb-2023-0156 37639730

[18] Bae TW Kim MS Park JW Kwon KK Kim KH . Multilayer perceptron-based real-time intradialytic hypotension prediction using patient baseline information and heart-rate variation. Int J Environ Res Public Health. (2022) 19:10373. doi: 10.3390/ijerph191610373 36012006 PMC9408052

[19] Schindele A Krebold A Heiß U Nimptsch K Pfaehler E Berr C . Interpretable machine learning for thyroid cancer recurrence predicton: Leveraging XGBoost and SHAP analysis. Eur J Radiol. (2025) 186:112049. doi: 10.1016/j.ejrad.2025.112049 40096773

[20] Khosravi P Fuchs TJ Ho DJ . Artificial intelligence-driven cancer diagnostics: enhancing radiology and pathology through reproducibility, explainability, and multimodality. Cancer Res. (2025) 85:2356–67. doi: 10.1158/0008-5472.CAN-24-3630 40598940

[21] Marzi S Vidiri A Ianiro A Parrino C Ruggiero S Trobiani C . CT-based radiomics models with external validation for prediction of recurrence and disease-specific mortality after radical surgery of colorectal liver metastases. Ann Surg Oncol. (2025) 32:9584–96. doi: 10.1245/s10434-025-18248-y 40928578

[22] Wang Q Nilsson H Xu K Wei X Chen D Zhao D . Exploring tumor heterogeneity in colorectal liver metastases by imaging: Unsupervised machine learning of preoperative CT radiomics features for prognostic stratification. Eur J Radiol. (2024) 175:111459. doi: 10.1016/j.ejrad.2024.111459 38636408

[23] Granata V Fusco R De Muzio F Brunese MC Setola SV Ottaiano A . Radiomics and machine learning analysis by computed tomography and magnetic resonance imaging in colorectal liver metastases prognostic assessment. Radiol Med. (2023) 128:1310–32. doi: 10.1007/s11547-023-01710-w 37697033

[24] Paro A Hyer MJ Tsilimigras DI Guglielmi A Ruzzenente A Alexandrescu S . Machine learning approach to stratifying prognosis relative to tumor burden after resection of colorectal liver metastases: an international cohort analysis. J Am Coll Surg. (2022) 234:504–13. doi: 10.1097/XCS.0000000000000094 35290269

[25] Giannini V Pusceddu L Defeudis A Nicoletti G Cappello G Mazzetti S . Delta-radiomics predicts response to oxaliplatin-based chemotherapy in colorectal cancer patients with liver metastases. Eur Congress Radiol. (2022) 14(1):241. doi: 10.26044/ecr2022/C-17769 35008405 PMC8750408

[26] Brunese MC Clemente A De Chiara M Nardone V Spiezia S Avella P . Radiomics of portal-phase ring enhancement: a novel imaging biomarker for bevacizumab response associated with overall survival rates. It might help with surgical decision-making in colorectal liver metastases? Updates Surg. (2026) 78:683–93. doi: 10.1007/s13304-025-02501-w 41606201 PMC13212809

[27] Li S Dai Y Chen J Yan F Yang Y . MRI-based habitat imaging in cancer treatment: current technology, applications, and challenges. Cancer Imaging. (2024) 24:107. doi: 10.1186/s40644-024-00758-9 39148139 PMC11328409

[28] Gan X Zhang W Shen Z Chen W Duan X Dai H . An interpretable machine learning model for predicting visceral pleural invasion in cT1 lung adenocarcinoma based on habitat analysis. Quant Imaging Med Surg. (2025) 15:12100–15. doi: 10.21037/qims-2024-2890 41367764 PMC12682543

[29] Shang Y Zeng Y Luo S Wang Y Yao J Li M . Habitat imaging with tumoral and peritumoral radiomics for prediction of lung adenocarcinoma invasiveness on preoperative chest CT: a multicenter study. AJR Am J Roentgenol. (2024) 223:e2431675. doi: 10.2214/AJR.24.31675 39140631

[30] Yuan J Wu M Qiu L Xu W Fei Y Zhu Y . Tumor habitat-based MRI features assessing early response in locally advanced nasopharyngeal carcinoma. Oral Oncol. (2024) 158:106980. doi: 10.1016/j.oraloncology.2024.106980 39151333

[31] Heiser CN Simmons AJ Revetta F McKinley ET Ramirez-Solano MA Wang J . Molecular cartography uncovers evolutionary and microenvironmental dynamics in sporadic colorectal tumors. Cell. (2023) 186:5620–5637.e16. doi: 10.1016/j.cell.2023.11.006 38065082 PMC10756562

[32] Wang F Long J Li L Wu ZX Da TT Wang XQ . Single-cell and spatial transcriptome analysis reveals the cellular heterogeneity of liver metastatic colorectal cancer. Sci Adv. (2023) 9:eadf5464. doi: 10.1126/sciadv.adf5464 37327339 PMC10275599

